# The Irrepressible Joker

**Published:** 1872-05

**Authors:** 


### THE IRREPRESSIBLE JOKER.

SOME years ago a little fellow out West wrote for medical advice. He had a great variety of complaints: he was too little; was humpbacked; had suffered from white swelling; hence one leg was shorter than the other, and one was longer than the other; the result was that he was lame, and nobody would marry him; besides these he had dyspepsia, was very diffident in the presence of young ladies, and the old ones he didn't fancy. Desiring something more definite, we wrote to know what his principal symptom was, the one which gave him the most annoyance. He replied, the greatest trouble of his life was that he

"HAD A HOLE IN HIS POCKET."

Not to be outdone by the good-natured stranger, we advised him to darn it up. He replied he didn't know how to darn. We directed him to learn.

"Well," said he, "I will learn; and more, I will learn to knit if I can get a knitting-machine. I did set about learning to keep books, spent all my money, and a great deal of time; but then I found the people were so poor out here in Indiana, that they have no books to keep. So I'm still at the bottom of the ladder. But I am going to learn how to knit, as I can't work, and am no beauty; but I'll make money, and then I can get a dozen wives."

Long years have passed since then, and we have often wondered what ever became of our joker; and this very day we have received a letter from him, saying, "My physical and pecuniary condition remain about the same. I make some fifty or sixty dollars a year by my knitting-machine; from which you will truly infer I am not ready to marry, and sometimes feel as forlorn as a

ROOSTER IN THE RAIN."

The man or woman who has the philosophy, the courage, and the ability to rise above the mishaps of life, to laugh off half its hardships, has a fortune within himself, equal in value to hoards of gold, which too often sadden the heart by every increase.

Here is a young man endeavoring to make an honest living for himself, in one of the humblest of human occupations, but as useful as it is honest; yet never having oppressed a fellow-creature in his need, nor wronged a human being of a farthing, nor owed a dollar, even if he did not own one, he sleeps soundly, and has a merrier heart by a thousand fold than many who live in their brown-stones, rustle in richest silks along the avenue, and drive their splendid turn-outs in the Park, either with the horrid incubus of debt, or the sharp-pointed memories of friends betrayed, of struggling industry ground to powder, of secret confidences forfeited, of damning deceptions practiced, of raising their own fortunes on the ruins of those who once trusted them wholly.

A lady with a young child, thousands of miles from home in a foreign land, fell short of money; expected remittances did not come; but appetite came every day, imperative and strong. It came to the point at last that she was obliged to borrow money from one of the servants in the house. But instead of being mortified to death at the humiliating position she was placed in, and crying like a baby, she absolutely made fun of her own predicament, and felt the braver and better of herself for not being either afraid or ashamed to borrow money of a menial.

That mother is of some account, and well merits the double title of philosopher and heroine.

